# Café Coronary Deaths: Medicolegal Observations From a Tertiary Care Center

**DOI:** 10.7759/cureus.98188

**Published:** 2025-11-30

**Authors:** Nagendra Singh Sonwani, Kishor Thakur, Navneet Ateriya, Arvind Kumar, Anil Kohli

**Affiliations:** 1 Forensic Medicine, Pt. Jawahar Lal Nehru Memorial Medical College, Raipur, IND; 2 Forensic Medicine, Government Medical College and Hospital, Kanker, IND; 3 Forensic Medicine and Toxicology, All India Institute of Medical Sciences, Gorakhpur, Gorakhpur, IND; 4 Forensic Medicine, University College of Medical Sciences and Guru Teg Bahadur (GTB) Hospital, Delhi, IND

**Keywords:** café coronary syndrome, food asphyxia, forensic autopsy, mechanical asphyxia, medicolegal

## Abstract

Introduction

Café coronary syndrome, or food asphyxia, is a sudden and unexpected death caused by upper airway obstruction by food during eating. Often mistaken for cardiac arrest, it commonly occurs in individuals who eat rapidly, are under the influence of alcohol, or have impaired swallowing reflexes due to neurological or dental issues.

Methods

A retrospective descriptive study was conducted on nine (N=9) medicolegal autopsies showing complete airway obstruction by food. Each case was examined for external and internal findings, toxicological results, and circumstantial evidence, including the type of food, site of obstruction, and levels of alcohol.

Results

All nine victims (eight males and one female, aged 25-57 years) died suddenly during or shortly after eating. Alcohol intoxication was detected in six (66.6%) cases, with blood levels ranging from 169 to 256.9 mg/100 mL. The obstructing food, mainly a bolus of chicken or a piece of meat, was found at the epiglottis or tracheal bifurcation, with congested, edematous lungs.

Conclusion

Café coronary syndrome is a preventable cause of sudden death, diagnosed after a meticulous autopsy. Scene correlation is a part of history taking, which itself is part of autopsy. Public education, avoidance of alcohol during meals, and awareness of the Heimlich maneuver are crucial for prevention.

## Introduction

Sudden death during a meal has long intrigued clinicians and forensic experts. While such cases are often mistaken for cardiac events, some result from airway obstruction by food, a condition termed café coronary syndrome. Introduced by Haugen in 1963, the term described sudden deaths in restaurants that mimicked heart attacks [1]. The true cause is asphyxia due to an impacted food bolus near the larynx. Early reports identified typical victims as middle-aged men with poor dentition, alcohol use, and large food intake [2], while later studies included elderly patients with neurological disorders, those on sedatives or antipsychotics, and children aspirating food particles [3-5].

The syndrome holds major clinical and forensic relevance, as external signs may be minimal and diagnosis relies on autopsy confirmation. The present study examines nine (N=9) autopsy-confirmed deaths from food asphyxia, emphasizing characteristic findings, risk factors, and the role of forensic investigation in determining cause and manner of death. The primary objective of this study is to evaluate forensic autopsy findings in deaths due to airway obstruction by food bolus (café coronary syndrome).

## Materials and methods

This retrospective descriptive study analyzed nine (N=9) medicolegal autopsies of sudden deaths occurring during or immediately after eating at a tertiary medical institute between August 2019 and July 2025, during which 6,978 autopsies were performed. Only cases with complete medicolegal and toxicological documentation and definitive airway obstruction by a food bolus were included, while deaths due to aspiration of vomitus, non-food foreign bodies, or natural diseases were excluded.

Differentiation of true café coronary obstruction from aspirated food particles was based on anatomical and morphological criteria: all included cases showed a single compact obstructing mass of unchewed or inadequately chewed food tightly wedged in the laryngeal inlet/upper trachea, causing complete luminal occlusion, whereas vomitus aspiration presents with diffuse particulate material scattered along bronchi without focal blockage. Autopsies followed standard forensic procedures, including external examination, airway dissection, and assessment of lungs for asphyxial changes.

Toxicological testing employed gas chromatography-flame ionization detection (GC-FID)/headspace gas chromatography for alcohol estimation and high-performance liquid chromatography (HPLC) for drug screening, ensuring quantitative reliability and minimizing artefacts from postmortem ethanol formation. Each case was analyzed for demographic, pathological, and toxicological correlations to identify contributory factors, especially alcohol intoxication and neurological impairment. Although the study was retrospective, all data were extracted from official medicolegal records and anonymized before analysis, ensuring responsible and ethical use of archived postmortem findings.

## Results

**Figure 1:**
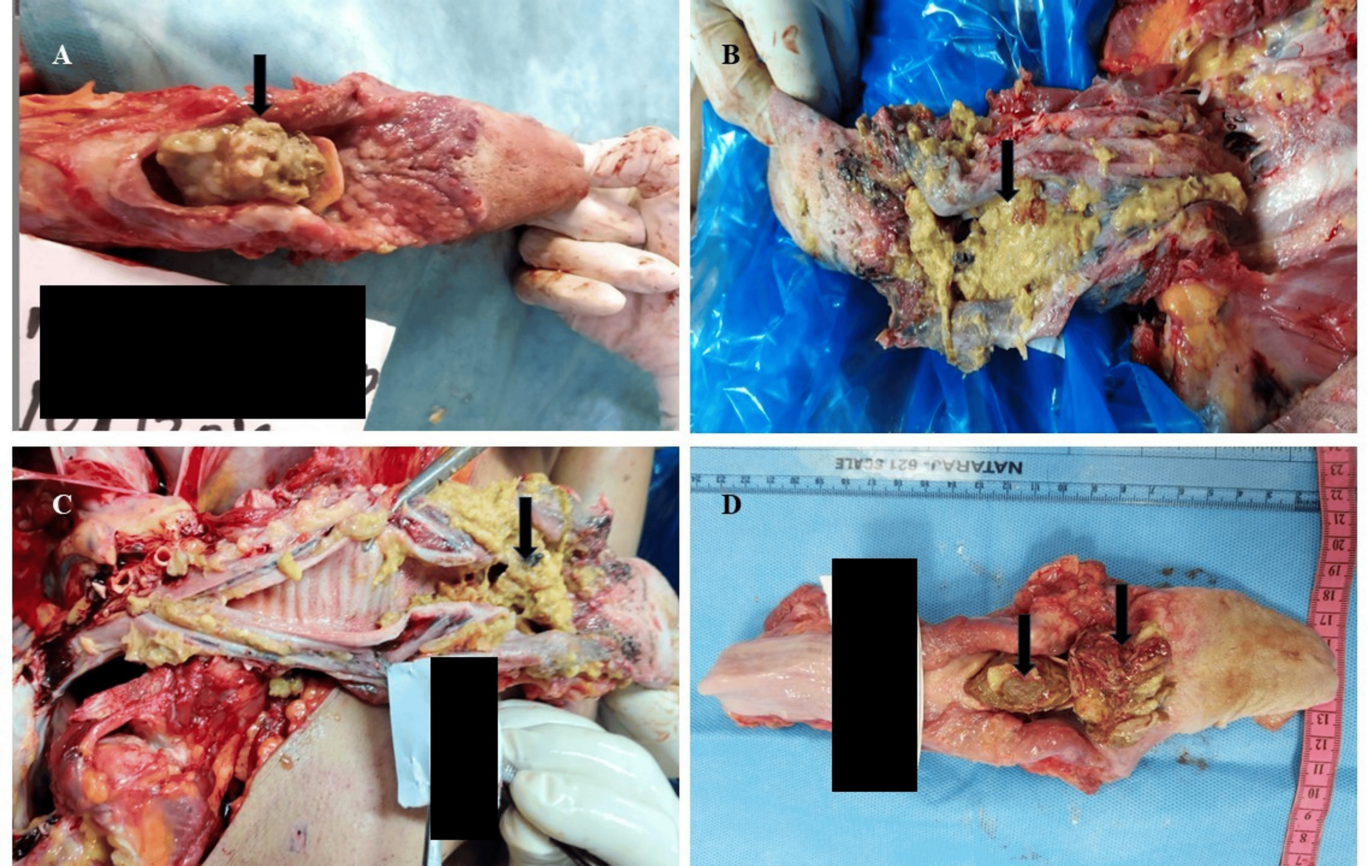
Food particles inside the respiratory passage (cases 1-4) A: Case 1 showing food particles mainly consisting of chicken. B: Case 2 showing food particles mainly consisting of rice. C: Case 3 showing food particles mainly consisting of chicken. D: Case 4 showing large boluses of chicken.

This paper presents nine (N=9) medicolegal autopsies of sudden deaths due to airway obstruction by food, classically known as café coronary syndrome. The first case involved a 45-year-old man who consumed alcohol and food at a roadside restaurant and was later found dead nearby. Autopsy revealed no external injuries. Although food stains were present around the mouth and nostrils, aspiration was ruled out because the autopsy revealed a single compact food bolus firmly impacted in the laryngeal inlet, causing complete obstruction. In aspiration deaths, diffuse food particles/gastric contents disperse into the bronchi, which was absent in this case. A large chicken piece (4.5 × 2.0 cm) obstructed the airway at the epiglottis, with another bolus near the tracheal bifurcation (Figure 1A). The lungs were congested, and the stomach contained semi-digested food similar to the bolus. Cardiac dissection revealed all major coronary arteries to be patent, with no evidence of thrombosis or significant atherosclerotic narrowing. Blood alcohol concentration was 256.9 mg/100 mL, indicating heavy intoxication. The cause of death was asphyxia due to choking, precipitated by alcohol-induced reflex suppression.

The second case was of a 52-year-old man who collapsed while eating dinner after prolonged alcohol consumption. Autopsy revealed conjunctival congestion, food particles in the mouth, and a contusion near the left eyebrow. Internally, the larynx was completely blocked by undigested rice (Figure 1B), and the liver showed changes of alcoholic cirrhosis. Cardiac dissection revealed all major coronary arteries to be patent, with no evidence of thrombosis or significant atherosclerotic narrowing. The blood alcohol level was 178 mg/100 mL. Death resulted from asphyxia due to food bolus obstruction, compounded by chronic alcoholism.

The third case concerned a 50-year-old man who collapsed after eating at a social event. Froth was seen at the mouth and nostrils. At autopsy, a piece of chicken was found obstructing the larynx (Figure 1C), and petechial hemorrhages were present on the lungs and heart. Cardiac dissection revealed all major coronary arteries to be patent, with no evidence of thrombosis or significant atherosclerotic narrowing. The stomach contained undigested food with an alcoholic odor, and the blood alcohol level was 92.8 mg/100 mL. The death was due to asphyxia from choking on chicken, with alcohol as a contributory factor.

The fourth case involved a 33-year-old man who suddenly collapsed near a bar-restaurant. Two large chicken pieces (4.6 × 3.0 cm and 4.9 × 4.0 cm) were found completely occluding the larynx (Figure 1D). Cardiac dissection revealed all major coronary arteries to be patent, with no evidence of thrombosis or significant atherosclerotic narrowing. The stomach contained rice and chicken with a strong alcoholic smell. The blood alcohol level was 169 mg/100 mL, and death was attributed to airway obstruction by a food bolus following alcohol ingestion.

The presence of food material in the respiratory passage in cases 1-4 is shown in Figure 1.

**Figure 2:**
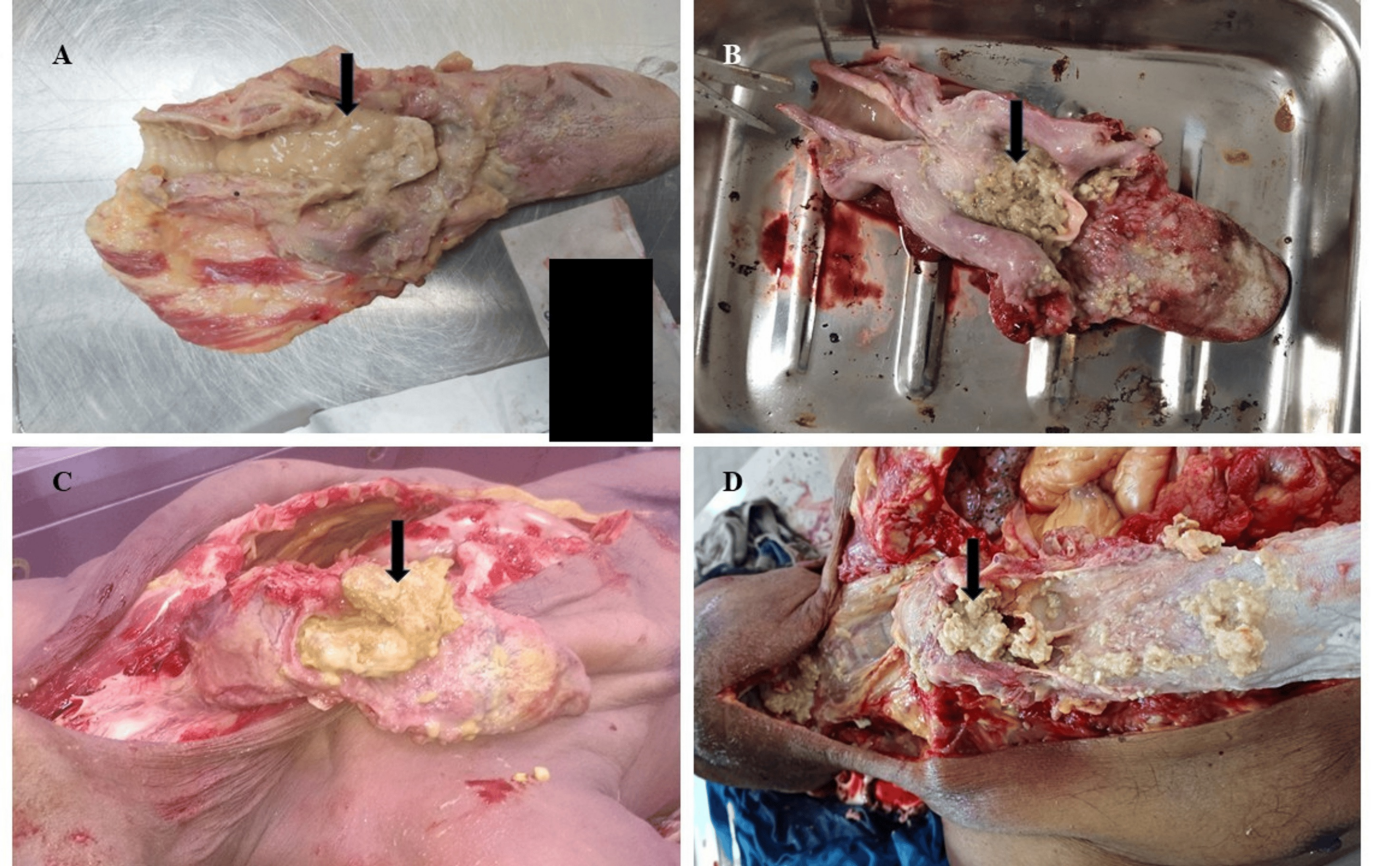
Food particles inside the respiratory passage (cases 5-8) A: Case 5 showing pasty food material. B: Case 6 showing mixed food particles. C: Case 7 showing food particles mainly consisting of meat. D: Case 8 showing food particles mainly consisting of meat.

The fifth case was a 57-year-old male prisoner with right-sided hemiparesis after a previous stroke. He collapsed during lunch in the prison ward. Autopsy showed cyanosis, but no injuries. A bolus of undigested food was found blocking the airway at the epiglottis (Figure 2A). Cardiac dissection revealed all major coronary arteries to be patent, with no evidence of thrombosis or significant atherosclerotic narrowing. The brain showed old hemorrhagic lesions, and toxicology was negative for alcohol or drugs. Death was due to choking by food, with neurological impairment acting as a predisposing factor.

In the sixth case, a 25-year-old female passenger died after a road accident. Although trauma was suspected, the autopsy showed no external or internal injuries. Instead, the airway was completely obstructed by semi-digested food particles (Figure 2B). The stomach contained similar material without an alcoholic odor. Cardiac dissection revealed all major coronary arteries to be patent, with no evidence of thrombosis or significant atherosclerotic narrowing. Toxicology was negative, and death was determined to be due to asphyxia from choking, unrelated to the collision.

The seventh case involved a 28-year-old man found unconscious with vomitus-stained clothes. Food debris was noted around the mouth, and a large meat bolus (5.2 × 3.0 cm) was found blocking the airway at the epiglottis (Figure 2C). The trachea was congested, and petechial hemorrhages were seen in the stomach lining. The stomach contained semi-digested food with an alcoholic odor, and the blood alcohol concentration was 128 mg/100 mL. Cardiac dissection revealed all major coronary arteries to be patent, with no evidence of thrombosis or significant atherosclerotic narrowing. Death was caused by asphyxia due to food choking under alcohol influence.

Case 8 was that of a 50-year-old man who collapsed while eating mutton at a street stall. A large meat piece (9 × 5 cm) was found occluding the upper airway (Figure 2D), and rice particles were seen around the nostrils. The lungs and heart showed petechial hemorrhages, and the stomach contained undigested food with an alcoholic odor. Cardiac dissection revealed all major coronary arteries to be patent, with no evidence of thrombosis or significant atherosclerotic narrowing. The blood alcohol level was 88 mg/100 mL. The cause of death was asphyxia due to choking by a meat bolus following alcohol consumption.

The presence of food material in the respiratory passage in cases 5-8 is shown in Figure 2.

The final case involved a 46-year-old man at a de-addiction center who collapsed while eating a banana. He was undernourished and had cyanosis, but no injuries. A small piece of banana was lodged in the vocal cords, while a larger fragment (4.7 × 2.9 cm) was impacted at the tracheal bifurcation. The image for case 9 could not be retrieved. Cardiac dissection revealed all major coronary arteries to be patent, with no evidence of thrombosis or significant atherosclerotic narrowing. Toxicology was positive for opioids. The cause of death was asphyxia due to choking by a banana, triggered by opioid-induced suppression of airway protective reflexes.

The detailed findings of all cases are summarized in Table 1.

**Table 1:** Summary of autopsy and toxicological findings in café coronary syndrome

Case	Age/sex	Food type	Site of obstruction	Toxicology
1	45 years/male	Chicken	Epiglottis and tracheal bifurcation	Blood alcohol: 256.9 mg/100 mL
2	52 years/male	Rice	Larynx	Blood alcohol: 178 mg/100 mL
3	50 years/male	Chicken	Larynx	Blood alcohol: 92.8 mg/100 mL
4	33 years/male	Chicken (2 pieces)	Epiglottis	Blood alcohol: 169 mg/100 mL
5	57 years/male	Pasty food	Epiglottis	Negative
6	25 years/female	Mixed food	Trachea	Negative
7	28 years/male	Meat	Epiglottis	Blood alcohol: 128 mg/100 mL
8	50 years/male	Meat	Oral cavity and airway	Blood alcohol: 88 mg/100 mL
9	46 years/male	Banana	Vocal cords and tracheal bifurcation	Positive for opioids

## Discussion

Choking refers to obstruction of the internal airway, usually between the pharynx and tracheal bifurcation, leading to interference with respiration and asphyxia [6]. Deaths from choking may be natural, homicidal, or accidental, with the latter being the most common in forensic practice [7]. Café coronary syndrome represents a distinct form of accidental choking, first described in the early 20th century. Puppe (1908) defined it as suffocation from large food pulp [8], while Kolisko (1913) attributed it to “laryngeal shock” from vagal inhibition. Although vagal inhibition has historically been proposed as a possible mechanism of sudden death during choking, contemporary forensic literature provides limited empirical support for its routine involvement. Most recent studies emphasize that mechanical airway obstruction remains the primary cause of collapse in café coronary cases, and evidence for fatal reflex cardiac arrest triggered by vagal stimulation is largely anecdotal. Therefore, while vagal inhibition cannot be entirely excluded as a contributory factor in rapid death, it should be regarded as a theoretical mechanism rather than a frequently proven cause [9]. Haugen (1963) later popularized the term to describe sudden, unexpected mealtime deaths caused by food bolus obstruction, often mistaken for coronary occlusion [1]. The term arose from restaurant incidents where victims collapsed suddenly while eating, giving the false impression of a cardiac event.

In this series of nine (N=9) medicolegal autopsies, all deaths were due to complete upper airway obstruction by food, confirming café coronary syndrome. Most victims were adult men aged 25-57 years, consistent with previous studies showing a male predominance [3,10,11]. The age range was younger than that reported in earlier literature (sixth to seventh decades) [10], possibly due to contributory factors such as alcohol intoxication, rapid eating, and poor mastication, recognized risks for choking [2,3,12,13].

Alcohol played a central role in six (N=6,66.6%) cases, with blood alcohol levels ranging from 88 mg/100 mL to 256.9 mg/100 mL. This finding aligns with prior studies identifying alcohol as the leading predisposing factor in food asphyxia. Alcohol depresses the central nervous system, blunting the gag and cough reflexes, delaying swallowing, and disrupting coordination between respiration and deglutition [3,13]. Saukko and Knight emphasized that choking deaths frequently follow ingestion of large, poorly chewed meat or chicken pieces, observations mirrored in the current study [14].

Rapid eating and poor dentition are also established risk factors [2,3,13]. Although not observed in every case, witness accounts indicated hurried ingestion. Most victims collapsed within moments of swallowing, reflecting the sudden and silent onset typical of café coronary deaths.

The mechanism of death is usually acute hypoxia from complete airway blockage. However, in some instances, reflex cardiac arrest due to vagal inhibition may cause near-instant death before visible asphyxial signs develop. Modern understanding suggests both mechanisms (mechanical obstruction and vagal inhibition) may coexist, with one predominating depending on the case [4,12]. The sudden collapse of several victims supports this dual mechanism theory.

Autopsy findings in all cases were consistent with complete airway obstruction, usually at the epiglottis or tracheal bifurcation. The trachea contained mucoid or frothy secretions, and lungs were congested and edematous, with pleural or epicardial petechiae in several cases. The obstructing food, mostly meat or chicken, occasionally rice or banana, varied in size, influencing both the impaction site and the speed of fatal asphyxia. Additionally, there was no blockade in the coronary arteries, which potentially excluded a cardiac cause. The mean alcohol concentration in this series (~152.12 mg/100 mL) was sufficient to impair reflexes but below the lethal intoxication threshold (~300 mg/100 mL).

In addition to alcohol, neurological and pharmacological impairments emerged as important contributory factors. One incarcerated individual with post-stroke hemiparesis died after choking on semi-solid food, supporting existing literature that links conditions such as cerebrovascular disease, dementia, and Parkinsonism with impaired swallowing reflexes and heightened choking vulnerability [3,13]. Another case (case 9) involved a 46-year-old drug rehabilitation patient who choked on a banana while under opioid influence, illustrating how sedatives and narcotics suppress swallowing and cough reflexes, predisposing to aspiration and obstruction.

External findings were typically minimal, food or froth near the mouth and nostrils, mild cyanosis, or no visible trauma. Such subtlety underscores the need to diagnose café coronary based on airway findings, toxicology, and circumstantial history rather than external clues [4]. Differentiating café coronary from aspiration of vomitus is essential. Gastroesophageal reflux disease (GERD) could not be definitively assessed or ruled out, as retrospective autopsy records do not routinely document premortem symptoms or endoscopic findings. However, no macroscopic features suggestive of chronic reflux injury, such as esophagitis, ulceration, or stricture, were noted in the available autopsy reports. The cause of death was established based on clear mechanical airway obstruction rather than lower esophageal pathology. In aspiration, acidic gastric contents reach the distal bronchi, whereas in café coronary, the obstructing bolus remains localized to the upper airway. A simple litmus test can confirm the acidity of aspirated material, aiding accurate postmortem interpretation.

All deaths in this series were accidental in nature, with no homicidal obstruction. However, in institutional environments, such as prisons or rehabilitation centers, issues of supervision and negligence may arise if high-risk individuals are left unattended during meals. Forensic pathologists must therefore document findings thoroughly to ensure defensible conclusions.

Café coronary syndrome remains a preventable cause of sudden death. Simple preventive measures, such as avoiding alcohol during meals, eating slowly, chewing food properly, and supervising neurologically impaired or intoxicated individuals, can drastically reduce risk. Public education on emergency maneuvers such as the Heimlich technique can save lives. Displaying instructional posters and conducting staff training in restaurants, hostels, and cafeterias should be encouraged [15].

The present study, although informative, has limitations. As a retrospective analysis of nine (N=9) autopsies, the sample size is small and may not represent broader trends. Detailed histopathology was unavailable for all cases, and the lack of scene investigation limited correlation with circumstantial findings. Variations in toxicological timing may have affected alcohol level interpretation. Additionally, data on dentition, swallowing disorders, or prior choking episodes were incomplete. Despite these limitations, the study reinforces the medicolegal and preventive importance of café coronary syndrome.

## Conclusions

In conclusion, this study reaffirms that café coronary syndrome, although relatively less prevalent, is a critical cause of sudden, unexpected death in forensic practice. The triad of heavy alcohol consumption, rapid eating, and impaired protective reflexes emerged as major risk factors. Accurate diagnosis requires meticulous autopsy, toxicological correlation, and contextual analysis, while awareness and prevention can avert such avoidable tragedies.
